# Analysis of molecular targets and mechanisms of Bisphenol F (BPF)-induced non-alcoholic fatty liver disease (NAFLD) based on network toxicology and molecular dynamics

**DOI:** 10.1371/journal.pone.0351730

**Published:** 2026-06-18

**Authors:** Riwei Wang, Qiangming Liao, Xianwei Liu, Liang Sun, Yun Xia

**Affiliations:** 1 Department of Three Branches of General Surgery, Jiujiang City Key Laboratory of Cell Therapy, JiuJiang NO.1 People’s Hospital, Jiujiang, China‌‌; 2 Department of Hepatobiliary and Pancreatic Surgery, the Second Affiliated Hospital, Jiangxi Medical College, Nanchang University, Nanchang, Jiangxi Province, China; 3 Department of Image Center, Jiujiang City Key Laboratory of Cell Therapy, JiuJiang NO.1 People’s Hospital, Jiujiang, China; Helwan University, EGYPT

## Abstract

Bisphenol F (BPF), a primary substitute for bisphenol A (BPA), is widely utilized in industrial production and daily life. However, its widespread environmental presence has raised concerns regarding potential health risks. This study aims to investigate the potential toxic targets of BPF in the pathogenesis of non-alcoholic fatty liver disease (NAFLD). Initially, potential target genes of BPF were identified using the ChEMBL, STITCH, and SWISS databases. NAFLD-related genes were obtained from the OMIM and GeneCards databases, yielding a preliminary set of 28 overlapping candidate targets. Gene Ontology (GO) and Kyoto Encyclopedia of Genes and Genomes (KEGG) enrichment analyses were subsequently performed to elucidate the biological processes and signaling pathways potentially affected by BPF. Differential expression analysis of transcriptomic data from NAFLD and normal liver tissues obtained from the GEO database (GSE260666) revealed that CYP2C19 and SHBG were significantly upregulated in NAFLD samples, suggesting their potential as key targets of BPF. Molecular docking simulations using AutoDock demonstrated stable binding conformations between BPF and both CYP2C19 and SHBG proteins, with favorable binding free energies indicating strong interactions. Furthermore, molecular dynamics simulations confirmed the structural stability of the protein-ligand complexes under simulated physiological conditions. These findings provide a theoretical basis for understanding the toxic targets and mechanisms of BPF in NAFLD pathogenesis and offer insights for the prevention and treatment of NAFLD associated with BPF exposure from plastic products.

## 1. Introduction

Non-alcoholic fatty liver disease (NAFLD) is a collective term for conditions characterized by abnormal lipid accumulation in the liver (hepatic steatosis) [1]. Simple fatty liver, without inflammation, is referred to as non-alcoholic fatty liver (NAFL), while non-alcoholic steatohepatitis (NASH) is defined as a more severe progression featuring inflammation and hepatocyte injury (steatohepatitis) [2]. Currently, one-quarter of the global population is affected by NAFLD [3]. While central obesity is predominant in North America and Europe (affecting ~83% of patients), a significant proportion of Asian patients exhibit “lean NASH” with a normal body mass index (BMI), despite Asia’s lower BMI cutoff for overweight classification (BMI > 23) [4]. By 2030, the prevalence of NAFLD in adults is projected to rise from 20% to 27% [5]. Globally, the adult prevalence of NAFLD was estimated at approximately 30% according to a recent systematic review [6]. Previous modeling studies projected an increase from 20% to 27% by 2030 based on historical trends [5]. Although steady progress has been made in elucidating NAFLD pathogenesis, identifying therapeutic targets, and advancing drug development, significant challenges remain. Therefore, curbing the rising NAFLD population has become an urgent global public health challenge, likely requiring environmental and lifestyle interventions.

Bisphenol A (BPA), an environmental endocrine-disrupting chemical (EDC), is primarily used in manufacturing food containers, epoxy resins, plastic tableware, polycarbonate plastics, and infant bottles [7]. Despite its widespread application in daily life, accumulating evidence has linked BPA exposure to various human diseases, including hepatic steatosis [8], type 2 diabetes [9], cardiovascular disorders [10], and abnormal liver enzymes [11]. Due to concerns over its ubiquitous exposure and potential adverse effects, BPA has been progressively banned and replaced by structurally analogous alternatives such as bisphenol S (BPS) and bisphenol F (BPF) [12,13]. Bisphenol F (BPF), a BPA substitute, chemically termed “4,4′-dihydroxydiphenylmethane”, is now extensively utilized in polycarbonate plastics, epoxy resins, structural adhesives, water pipes, and food-contact materials [14]. However, its pervasive use has led to detectable levels of BPF in diverse environmental matrices, foodstuffs, and bodily fluids [15,16], indicating multiple human exposure routes. Despite its widespread adoption, the potential health risks of BPF remain poorly understood. Current evidence suggests that certain environmental chemicals may disrupt adipose tissue energy metabolism and endocrine regulation [17]. As a lipophilic compound, BPA impairs mitochondrial function and induces oxidative stress, promoting lipid accumulation and subsequent NAFLD-like pathological changes [18]. Given the structural similarity between BPF and BPA, BPF may exert analogous effects on adipocytes. Emerging studies associate BPF exposure with reproductive dysfunction, hepatic injury, and metabolic syndrome [19–21].

The liver, as one of the primary target organs of bisphenols, has been extensively studied in recent years, with mounting evidence indicating that bisphenol exposure induces hepatic metabolic dysfunction [22,23]. Notably, studies have demonstrated the lipotoxic effects of BPF, which promotes the accumulation of lipid droplets (LDs) in hepatocytes, leading to NAFLD-like pathological alterations [24]. However, the underlying mechanisms remain largely elusive. Given the structural similarity between BPF and BPA—and its potential for equal or greater biological activity—BPF has been increasingly detected in global environmental matrices, food products, and human biological samples. This widespread presence raises concerns about its potential adverse effects on public health and the environment. Nevertheless, the precise molecular mechanisms underlying its toxicity and biological impact remain unclear. To address this knowledge gap, we employed network toxicology and molecular docking approaches to systematically identify potential targets and elucidate the molecular mechanisms by which BPF contributes to NAFLD pathogenesis.

## 2. Methods

### 2.1. Toxicity network analysis and structural characterization of BPF

The integration of network search algorithms and biological toxicity prediction methods into specialized software tools enables toxicity prediction of BPF compounds using structural modeling approaches. ADMETlab (https://admet.scbdd.com/), a comprehensive database dedicated to systematic drug evaluation, provides extensive data support for pharmaceutical research by covering absorption, distribution, metabolism, excretion, and toxicity (ADMET) properties. Using this platform, we performed systematic prediction and evaluation of BPF’s environmental toxicity profiles. For structural analysis, we obtained the canonical SMILES notation, 2D molecular structure, and optimized 3D conformation of BPF (PubChem CID: 12111) from the PubChem database (https://pubchem.ncbi.nlm.nih.gov/). These structural data were subsequently prepared for advanced computational analyses, including molecular docking and quantitative structure-activity relationship (QSAR) modeling.

### 2.2. Construction of interaction network and functional enrichment analysis of BPF-related target genes

Using the obtained SMILES information, we systematically screened potential target genes of BPF through three established databases: ChEMBL (https://www.ebi.ac.uk/chembl/), STITCH (http://stitch.embl.de/), and SwissTargetPrediction (http://www.swisstargetprediction.ch/). For ChEMBL and STITCH, we applied their default high-confidence score thresholds (ChEMBL: pChEMBL value > 5; STITCH: combined score > 0.7). For SwissTargetPrediction, we selected targets with associated binding probabilities. Protein-protein interaction networks of these candidate genes were constructed using STRING database. The resulting network data were imported into Cytoscape (version 3.10.3) for comprehensive network visualization and topological parameter calculation, including degree centrality and betweenness centrality. Bioinformatics analysis was subsequently performed using R software with clusterProfiler, org.db, and ggplot2 packages for Gene Ontology (GO) and Kyoto Encyclopedia of Genes and Genomes (KEGG) pathway analyses.

### 2.3. Acquisition of NAFLD-associated genes

The NAFLD-related genes were systematically retrieved from multiple authoritative databases, including: OMIM (Online Mendelian Inheritance in Man) database and GeneCards database. The search strategy employed the following key terms: “non-alcoholic fatty liver disease”, “NAFLD”. Duplicate entries were removed and gene symbols were standardized using HUGO Gene Nomenclature Committee (HGNC) guidelines. The final gene set was further validated against recent NAFLD-related publications in PubMed to ensure comprehensiveness.

### 2.4. Screening and enrichment analysis of core target genes

To further identify the core targets of BPF affecting NAFLD, we performed a homology comparison between the BPF related target genes obtained above and NAFLD-related action genes, and determined that BPF may cause NAFLD through these gene sets. Then the interconnection information between these genes is obtained from the STRING database, and the obtained result file is imported into Cytoscape_v3.10.3 for visualization and calculation of various parameters of each node. Gene ontology (GO) terms and Kyoto Encyclopedia of Genes and Genomes (KEGG) pathway analysis were performed on these genes to explore their possible biological pathways.

### 2.5. Acquisition and processing of NAFLD-related sequencing data

We initially acquired the NAFLD-related dataset GSE260666 (Illumina NovaSeq 6000 Homo sapiens) from the GEO database (https://www.ncbi.nlm.nih.gov/geo/), which contained sequencing data from 5 normal liver samples and 9 NAFLD samples. Using GEO2R analysis, we identified differentially expressed genes (DEGs) with the following stringent criteria: Adjusted p-value (Padj) < 0.05, Log2 fold change threshold > 1 (S1 Table). These DEGs were then cross-referenced with our previously identified hub genes to obtain a final set of differentially expressed core genes. This intersectional gene set was selected as the primary focus for subsequent investigations.

### 2.6. Molecular docking of BPF to key targets

After preliminary identification of key BPF targets, molecular docking simulations were performed to investigate intermolecular interactions and binding patterns between BPF and target proteins. The 2D structure of BPF was obtained from PubChem database (http://pubchem.ncbi.nlm.nih.gov/) and converted to 3D structure using ChemOffice software, then saved as mol2 file format. High-resolution crystal structures of target proteins were retrieved from RCSB PDB database (http://www.rcsb.org/) and prepared using PyMOL software, including removal of water molecules and phosphate groups, before saving as PDB files. Protein and ligand structures were processed using AutoDock tools, including addition of hydrogen atoms, removal of water molecules, and determination of rotatable bonds for the ligand. Docking grid box coordinates were then defined. Molecular docking was performed using AutoDock Vina 1.1.2 software to explore protein-ligand interactions. The optimal binding conformation was selected based on docking scores. Finally, PyMOL and Discovery Studio 2019 software were used for visualization and analysis of interactions between BPF and key amino acid residues. The specific grid center coordinates and the dimensions of the boxes for SHBG and CYP2C19 used for replication will be presented in S2 Fig.

### 2.7. Molecular dynamics simulation

Molecular dynamics simulations were performed using GROMACS 2022. Force field parameters were obtained through GROMACS’s pdb2gmx tool and the AutoFF website. During the simulation, the CHARMM36 force field was employed for receptor protein parameters, while the CGenFF force field was used for ligand parameters. The system was solvated with a 1 nm cubic TIP3P water box. Ions were added to the system using the gmx genion tool to achieve electrical neutrality. Long-range electrostatic interactions were processed using the Particle Mesh Ewald (PME) method with a cutoff distance of 1 nm. All bond constraints were implemented via the SHAKE algorithm, and the Verlet leap-frog algorithm was adopted with an integration time step of 1 fs during molecular dynamics simulations. Prior to molecular dynamics simulation, the system underwent energy minimization. The minimization process consisted of 3000 steps of steepest descent optimization followed by 2000 steps of conjugate gradient optimization. The optimization procedure included: first constraining the solute while minimizing water molecules; then constraining counterions during minimization; and finally performing full-system minimization without constraints. Simulations were conducted under NPT ensemble conditions at 310 K for 100 ns. During the simulation, gmx rmsd, gmx rmsf, gmx hbond, gmx gyrate, and gmx sasa tools were used to calculate root-mean-square deviation (RMSD), root-mean-square fluctuation (RMSF), hydrogen bonds (HBonds), radius of gyration (Rg), solvent accessible surface area (SASA), and Gibbs free energy, respectively. The MM-PBSA binding free energy of complexes was calculated using the gmx_MMPBSA package in GROMACS.

## 3. Results

### 3.1. The overall conceptual diagram of the research

**Fig 1:**
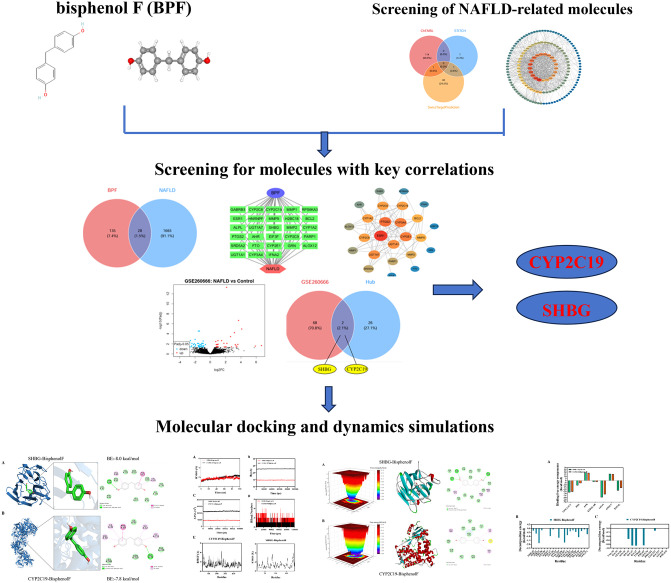
Research Roadmap‌‌.

The general research approach is as follows (Fig 1):

### 3.2. The structure of BPF

**Fig 2:**
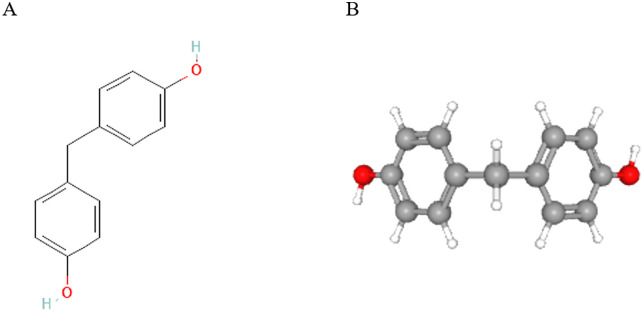
The Structure of BPF. **(A)** 2D Structure;**(B)** 3D Conformer.

**Fig 3:**
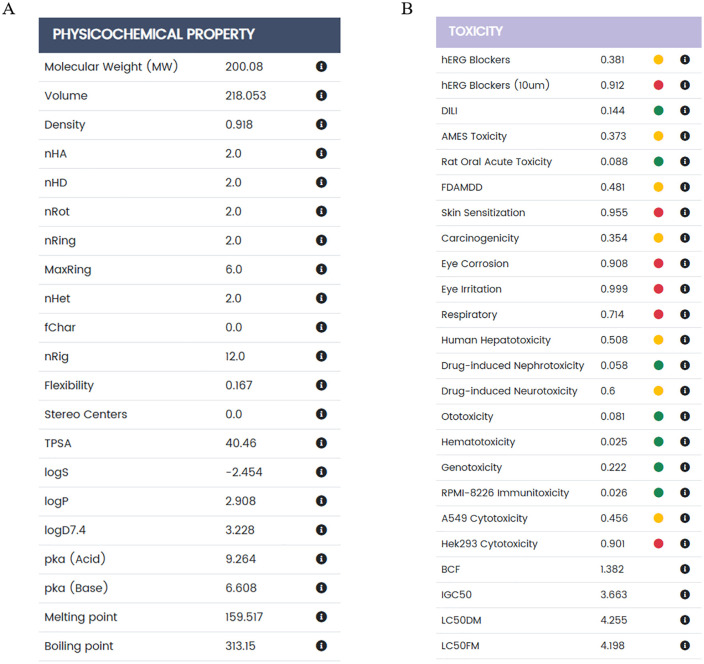
BPF Toxicity Analysis. **(A)** Physicochemical information of BPF; **(B)** Toxicity prediction analysis of BPF on various human organs.

We initially retrieved the chemical structure of BPF from the PubChem database. The canonical SMILES notation was obtained, along with the 2D structural representation (Figs 2A) and 3D conformational data (Fig 2B). These structural files were subsequently prepared for computational analysis.

### 3.3. Preliminary network assessment of BPF toxicity

Through the output results of the ADMETlab 3.0 software tool, we obtained the summary of the BPF toxicity model. For each endpoint, a prediction score (usually a probability value) and a classification result (such as “toxic/non-toxic”) were generated. The specific scores are listed in S1 Fig. This toxicity model indicates that the activity toxicity is related to human liver damage, and these findings are consistent with the previous reports in the literature regarding the toxic effects of BPF in humans. This lays the foundation for our further systematic and in-depth study of the toxicity effects of BPF on the human body (Fig 3).

### 3.4. Acquisition and enrichment analysis of BPF target genes

**Fig 4:**
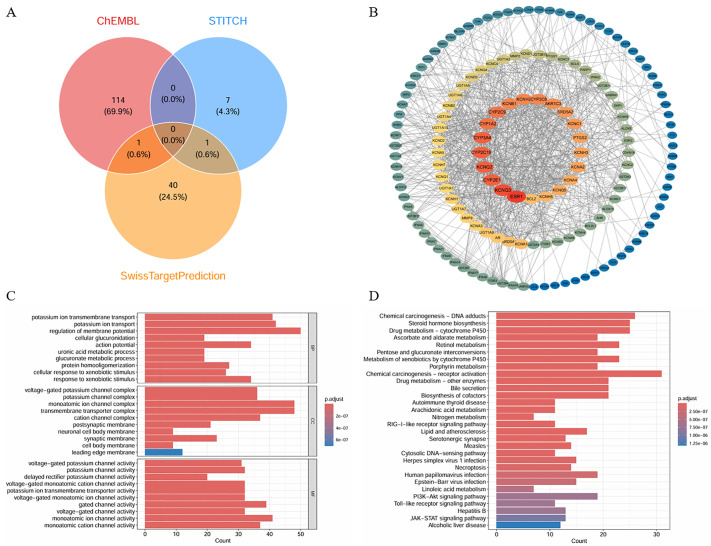
Screening of BPF target genes and functional enrichment. **(A)** Venn diagram of BPF target genes predicted by three databases; **(B)** The 163 BPF target genes were visualized by Cytoscape software. **(C)** GO enrichment analysis of target genes; **(D)** KEGG enrichment analysis of target genes‌‌.

A total of 163 potential BPF target genes were screened from ChEMBL, STITCH and SwissTargetPrediction databases. Then the PPI protein interaction network of BPF target genes was constructed using String database and visualized using Cytoscape software (Fig 4A, B). Subsequently, to further analyze the biological pathways that these genes may be involved in, GO and KEGG analyses were performed on these genes. KEGG results showed that BPF target genes may be related to chemical carcinogenesis, drug metabolism, and bile secretion (Fig 4C, D).

### 3.5. Target screening and enrichment analysis of BPF affecting NAFLD

**Fig 5:**
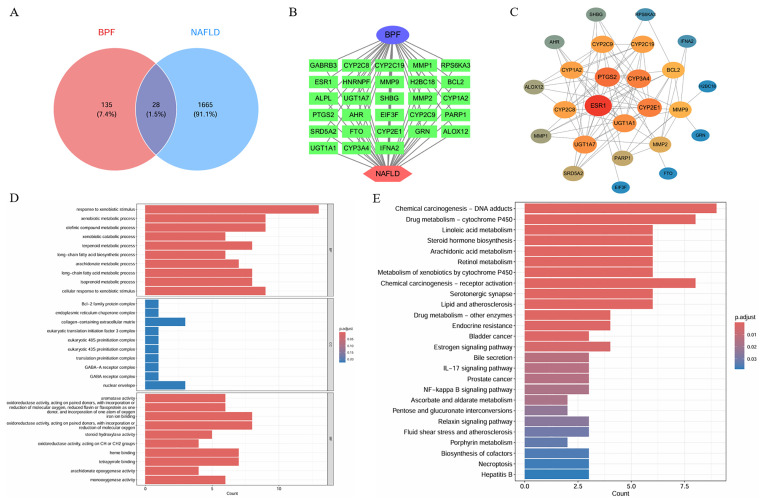
Constructing the BPF-target-NAFLD protein interaction network. **(A)** Veen map of crossover between BPF genes and NAFLD-related target genes; **(B, C)** interaction network of core genes; **(D, E)** GO and KEGG enrichment analysis of core genes‌‌.

In this study, we initiated a comprehensive screening process that initially identified 163 potential BPF-related targets. Subsequently, using available data from GeneCards and OMIM databases, we determined 1,693 targets closely associated with NAFLD. By integrating these two gene sets, we narrowed our focus to a core set of 28 overlapping targets, representing potential candidate targets for BPF’s effects on NAFLD. The Venn diagram illustrates these core targets shared between BPF and NAFLD (Fig 5A). We then constructed a BPF-target-NAFLD protein interaction network using the STRING database and visualized it with Cytoscape software (Fig 5B, C). Functional enrichment analysis (GO and KEGG) was performed on these 28 core genes. The GO enrichment analysis revealed that the core genes were primarily involved in responses to foreign stimuli, cellular responses to exogenous stimuli, and xenobiotic metabolic processes (Fig 5D). KEGG analysis indicated that the core genes were associated with pathways including chemical carcinogenesis, drug metabolism, and steroid hormone biosynthesis (Fig 5E).

### 3.6. NAFLD-related sequencing data were used to screen core differential genes

**Fig 6:**
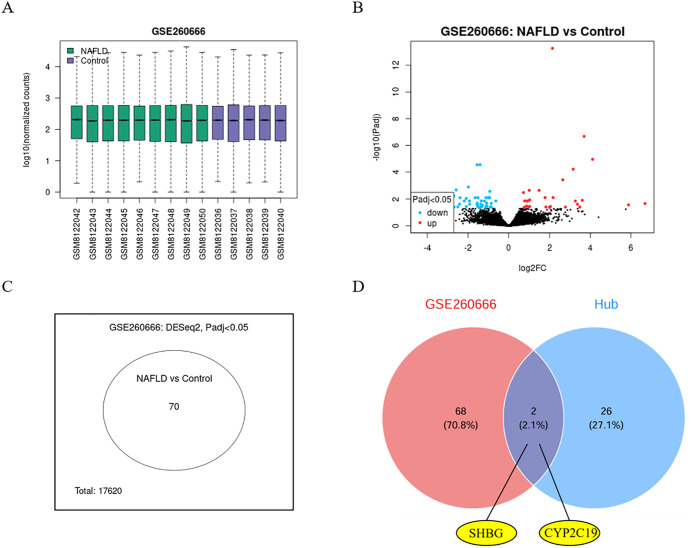
Screening key differentially expressed genes using the GEO database. **(A)** Homogenization of 14 sample data; **(B)** differentially expressed genes between normal liver and NAFLD Volcano diagram; **(C)** 70 differentially expressed genes that met our prespecified criteria were screened out; **(D)** Two core genes SHBG and CYP2C19 with differential expression were screened out‌‌.

To further identify core genes with differential expression, we analyzed NAFLD sequencing data from the GEO database (GSE260666) using the following criteria: P adj < 0.05 and Log2 fold change threshold>0. The dataset included 5 control samples (normal liver tissue group) and 9 NAFLD samples (4 NASH and 5 NAFL cases) (Fig 6A). Through analysis with the online GEO2R tool, we identified 70 differentially expressed genes meeting these criteria (Fig 6B, C). Subsequent integration of these differentially expressed genes with our previously screened core genes yielded two differentially expressed core genes: SHBG and CYP2C19 (Fig 6D).

### 3.7. Molecular docking of BPF with NAFLD core target proteins

**Fig 7:**
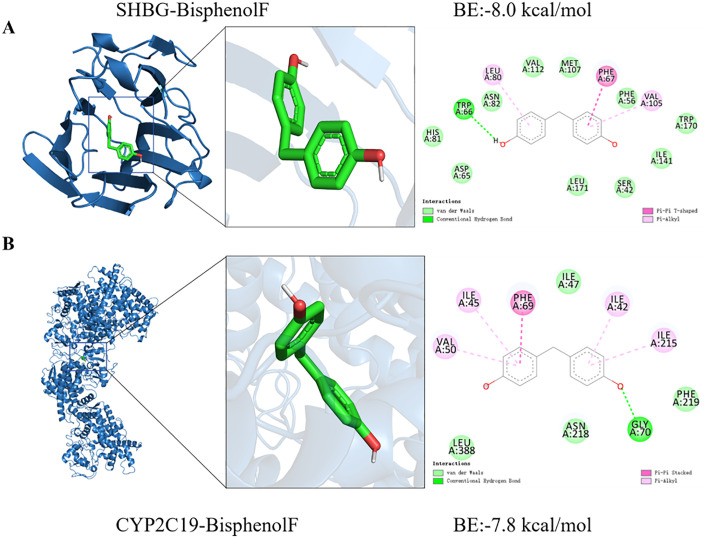
Demonstration of molecular docking results. **(A)** Demonstration of the docking results of SHBG and BPF; **(B)** Display of the docking results of CYP2C19 and BPF.

In general, a binding energy <0 kcal/mol indicates spontaneous binding between receptor and ligand without external energy input; < −5.0 kcal/mol suggests good binding affinity; while <−7.0 kcal/mol demonstrates strong binding activity. Lower binding energy values correspond to stronger binding activity, higher affinity, and more stable conformations [25]. Our results demonstrated excellent docking performance, revealing strong binding capability between BPF and both SHBG and CYP2C19, highlighting their potent affinity and crucial role in BPF’s molecular mechanism affecting NAFLD. To further elucidate the complex binding configurations, we visualized the lowest-energy binding conformations using PyMOL (Fig 7A, B). For comparative purposes, we present the binding mode and binding free energy of SHBG with its known ligand, dihydrotestosterone (S3 Fig).

For SHBG: hydrogen bond interaction with TRP66; van der Waals interactions with ASN82, VAL112, MET107, PHE56, TRP170, ILE141, SER42, LEU171, ASP65 and HIS81; Pi-Pi T-shaped interaction with PHE67; and hydrophobic interactions with LEU80 and VAL105. For CYP2C19: hydrogen bond interaction with GLY70; van der Waals interactions with LEU388, ASN218, PHE219 and ILE47; Pi-Pi T-stacked interaction with PHE69; and hydrophobic interactions with VAL50, ILE45, ILE42 and ILE215. Both SHBG and CYP2C19 exhibited strong binding activity with BPF. We have placed all the binding affinities, including different docking poses and corresponding scores, in S4 and S5 Figs.

### 3.8. Molecular dynamics simulation analysis

Since semi-flexible docking cannot account for protein flexibility, temperature, pressure, or solvent effects, molecular dynamics (MD) simulations were performed on SHBG and CYP2C19 complexes with BPF to further validate binding stability. The co-crystallized ligands of each protein served as positive controls for comparative stability analysis.

**Fig 8:**
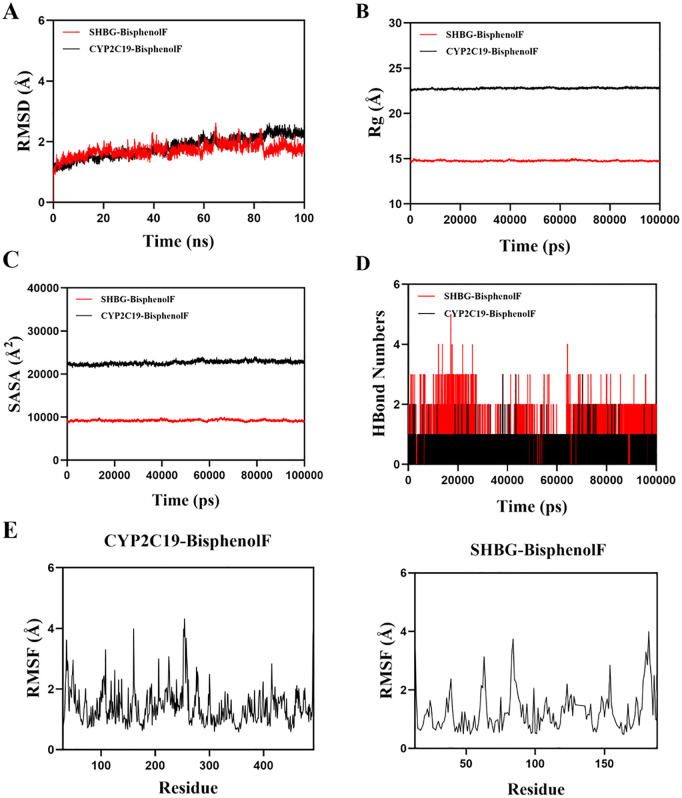
Molecular Docking dynamics simulation. **(A)** represents RMSD curve. **(B)** shows Rg curve; **(C)** represents SASA Curve; **(D)** represents the fluctuation curve of the number of hydrogen bonds; **(E)** represents the RMSF curve.

The root mean square deviation (RMSD) serves as a robust metric for evaluating the conformational stability of both proteins and ligands, quantifying the positional deviation of atoms from their initial coordinates. Lower RMSD values correspond to greater conformational stability. We employed RMSD analysis to assess system equilibration during simulations. As illustrated in Fig 8A: The SHBG-BPF complex achieved equilibrium after 85 ns, maintaining stable fluctuations within ±1.7 Å; The CYP2C19-BPF complex reached equilibrium within 5 ns, with subsequent fluctuations constrained to ±2.3 Å. These observations demonstrate the high binding stability of bisphenol F (BPF) with both target proteins, SHBG and CYP2C19.

The radius of gyration (Rg) serves as a quantitative measure for evaluating global structural changes and characterizing the compactness of protein structures. Larger Rg variations indicate greater system expansion. As shown in Fig 8B, both the SHBG-BPF and CYP2C19-BPF complexes exhibited stable Rg fluctuations throughout the simulation trajectory, demonstrating that the protein-ligand complexes maintained their structural integrity without significant expansion or contraction during dynamic motion.

SASA serves as a key metric for evaluating protein surface area. In this study, we calculated the SASA between target proteins and ligands (Fig 8C). The results demonstrated minimal changes in SASA for both the SHBG-BPF and CYP2C19-BPF complexes following ligand binding, indicating that BPF incorporation had negligible effects on protein structural conformation.

Hydrogen bonds play an important role in the binding of ligands to proteins. The number of hydrogen bonds between the small molecule and the target protein during the kinetic process is shown in Fig 8D. The number of hydrogen bonds between the SHBG-BisphenolF small molecule and the target protein ranges from 0 to 5, and in most cases, the complex has about 2 hydrogen bonds. The number of hydrogen bonds between the CYP2C19-BisphenolF small molecule and the target protein ranged from 0 to 3, and in most cases the complex had approximately 1 hydrogen bond. This suggests that this ligand has a good hydrogen bond interaction with the target protein.

Root mean square fuctuation (RMSF) can represent the flexibility of amino acid residues in a protein. As shown in Fig 8E, the RMSF values of SHBG-BisphenolF and CYP2C19-BisphenolF complexes are relatively low, so they are less flexible and more stable.

### 3.9. Free energy display between molecular docking

**Fig 9:**
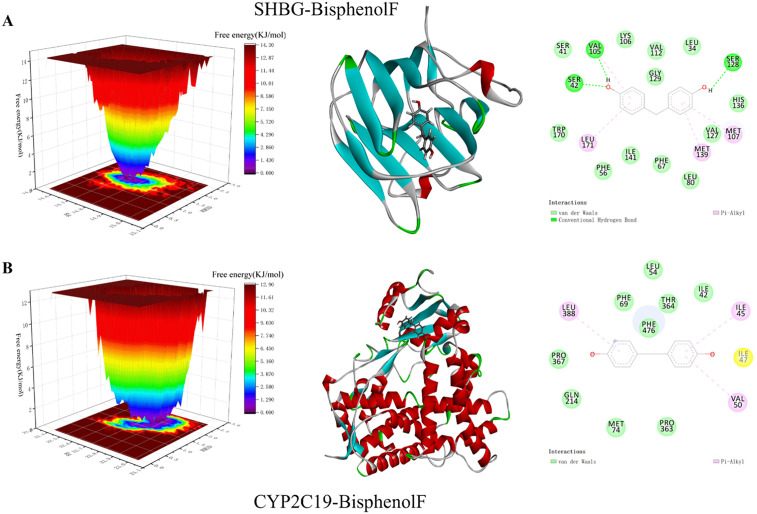
Visualize and display the strength of protein-ligand interaction. **(A)** Display of the binding free energy of SHBG and BPF; **(B)** Display of the binding free energy of CYP2C19 and BPF‌‌.

The free energy landscape (FEL) uses a visual representation of free energy changes to explore the molecular energy landscape and protein-ligand interactions, with energy minima representing steady states and maxima representing obstacles to conformational changes. This approach enables prediction of ligand binding affinity and elucidation of molecular recognition mechanisms. In the conformation where the energy minimum appears in the SHBG-BisphenolF complex (Fig 9A), SER41, LYS106, VAL112, GLY129, LEU34, HIS136, VAL127, LEU80, PHE67, ILE141 and PHE56 formed van der Waals interactions with small molecules on the receptor. VAL105, SE42, and SER128 on the receptor form Conventional Hydrogen Bond interactions with small molecules. In addition, LEU171, MET139, and MET107 exhibit Pi-Alkyl interactions with small molecules. In the conformation where the energy minimum appears in the CYP2C19-BisphenolF complex (Fig 9B), LEU54, PHE69, PHE476, YHR364, ILE42, ILE47, PRO363, MET74, GLN214 and PRO369 formed van der Waals interactions with small molecules on the receptor. LEU388, ILE45, and VAL50 on the receptor exhibit Pi-Alkyl interactions with small molecules.

**Fig 10:**
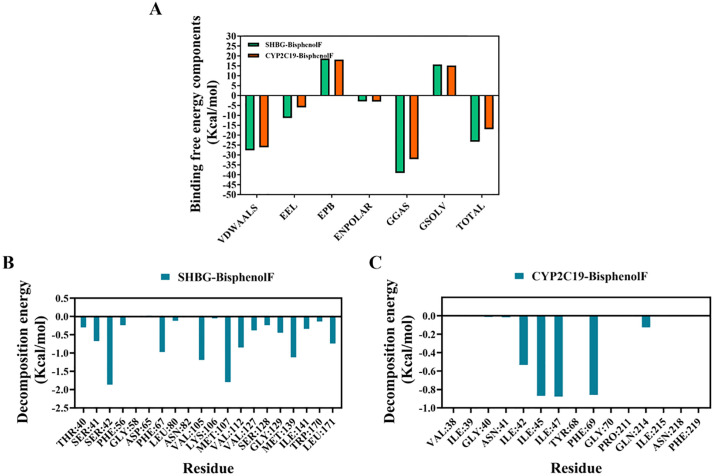
Based on the conformational display of ligand binding and the free energy of binding. **(A)** The binding free energy between SHBG and CYP2C19 and BPF was calculated by MM/PBSA method; **(B)** amino acids that contribute significantly to the binding of SHBG to BPF; **(C)** amino acids that contribute significantly to the binding of CYP2C19 to BPF‌‌.

The binding free energies of the complexes were calculated using MM/PBSA method based on their binding conformations (Fig 10A). The SHBG-BPF and CYP2C19-BPF complexes exhibited binding free energies of −5.57 kcal/mol and −4.06 kcal/mol, respectively. The negative values indicate binding affinity of the molecule to target proteins, with lower values representing stronger binding. Thus, the SHBG-BPF complex showed higher affinity. Key contributing residues were further analyzed: in SHBG-BPF complex, SER42, MET107, VAL105, MET139 and PHE67 showed high contribution values (Fig 10B); in CYP2C19-BPF complex, ILE47, ILE45, PHE69 and ILE42 demonstrated high contribution values (Fig 10C), suggesting these residues may play important roles in catalytic processes. (1 kcal/mol ≈ 4.184 kJ/mol）

In conclusion, the SHBG-BisphenolF and CYP2C19-BisphenolF complex system bound stably and had low binding free energy. Therefore, BisphenolF small molecules may act by inhibiting SHBG and CYP2C19 target proteins.

## 4. Discussion

Bisphenols are extensively utilized in modern industrial and consumer products [26]. BPA-based polycarbonate plastics are particularly valued for their lightweight, transparent, colorable, impact-resistant, heat-resistant, and chemically durable properties. These characteristics led to BPA becoming one of the most widely produced and used chemicals globally, primarily in manufacturing food containers, epoxy resins, plastic tableware, polycarbonate plastics, and infant bottles [7]. However, growing evidence linking BPA to various human diseases has prompted industrial shifts toward safer alternatives. Currently, BPF and other bisphenol analogues—such as bisphenol S (BPS) and bisphenol F (BPF) are increasingly adopted as BPA substitutes in industrial production [13].

BPF, with the chemical formula (HOC₆H₄)₂CH₂, is structurally analogous to bisphenol A (BPA). Marketed as a purportedly safer alternative to BPA, BPF has seen widespread commercial and industrial adoption. However, environmental monitoring reveals comparable detection frequencies and concentrations of BPF and BPA in indoor environments, food products, and human biomonitoring studies [27]. Emerging evidence suggests BPF’s health risks may parallel or exceed those of BPA. Studies associate BPF exposure with: Neurological dysfunction [28], Reproductive system impairments [29], Hyperglycemia [22], Hepatic lipid metabolism disorders [20]. Notably, recent research demonstrates that BPF promotes lipid droplet accumulation in hepatocytes via mitochondrial dysfunction, inducing NAFLD-like pathological changes [30]. These findings warrant urgent investigation into whether BPF contributes to the rising global incidence of NAFLD.

In China, the prevalence of NAFLD has continued to increase over the past 20 years, and NAFLD is currently the most common liver disease with a prevalence as high as 32.9% [31]. In recent years, more and more studies have found that environmental risk factors, especially environmental endocrine interruptors (EDCs), play a crucial role in the occurrence and development of nonalcoholic fatty liver disease (NAFLD) [32]. Due to the widespread use of BPA in the past, we found its pathogenic effect on NAFLD, and it has been widely banned. However, with the use of its substitute BPF, some studies have found that BPF exposure can also cause non-alcoholic fatty liver disease (NAFLD) -like changes [30]. Therefore, it is necessary to further explore the pathogenesis of NAFLD caused by BPF.

The advancement of high-throughput sequencing methodologies, molecular docking simulations, and comprehensive bioinformatics has provided robust tools for investigating compound toxicity mechanisms and enabling targeted risk assessment. In this study, we employed structural analysis of BPF combined with multi-database screening to predict its potential target genes, ultimately identifying two differentially expressed core genes: SHBG and CYP2C19. SHBG, a homodimeric glycoprotein synthesized by hepatocytes under hormonal and nutritional regulation [33], is secreted into circulation where it binds sex steroids with high affinity to modulate their bioavailability [34]. Beyond its classical role, SHBG is now recognized as a hepatokine actively participating in metabolic disease pathogenesis. Emerging epidemiological evidence identifies hepatic fat content—rather than systemic or visceral adiposity—as the primary determinant of circulating SHBG levels [35]. Recent clinical research demonstrates SHBG’s functional involvement in NAFLD development through modulation of hepatic lipogenesis, rather than merely serving as a passive biomarker [36]. Experimental studies using in vitro and in vivo models further reveal that SHBG overexpression may protect against NAFLD progression by regulating key lipogenic enzymes [37]. More and more studies have shown that SHBG itself, as a liver factor, plays an important biological role in the progression of NAFLD. Taken together with our study, it is more plausible that BPF may affect the progression of NAFLD through SHBG, and SHBG can be a potential biomarker for predicting the progression of NAFLD, as well as a novel preventive and therapeutic target for NAFLD.

CYP2C19, a major hepatic drug-metabolizing enzyme, plays pivotal roles in the activation and clearance of therapeutic agents, endogenous biomolecules, and environmental toxicants. Clinical studies have demonstrated significant downregulation of CYP2C19 in NAFLD patients, establishing its close association with disease progression [38]. Recent investigations further reveal that CYP2C19 mRNA and protein levels decline to approximately 40% of normal values during metabolic dysfunction-associated steatotic liver disease (MASLD) progression, with evidence suggesting this reduction occurs as early as the MASH (metabolic dysfunction-associated steatohepatitis) stage [39]. These studies all highlight the role of CYP2C19 in the progression of NAFLD and are in line with our results through multi-database screening and analysis. Follow – up studies increases the reliability of our findings and the feasibility of follow-up studies.

In conclusion, the two key genes we identified, SHBG and CYP2C19, play crucial roles in the progression of NAFLD. At present, relevant studies have shown their potential mechanisms in regulating the progression of NAFLD, supporting their importance. Therefore, our study showed that the compound BPF may affect the progression of NAFLD through these two key genes. The interaction of BPF with SHBG and CYP2C19 proteins was investigated by molecular docking technology, and the results showed that the binding energy of BPF to them was low, implying that BPF could firmly bind to them. One of the key directions for future research is to systematically compare the binding affinity and binding patterns of BPF with those of other endocrine-disrupting substances with toxicological relevance (particularly substances like BPS, which are bisphenol analogues) at these key targets (such as SHBG and CYP2C19) and other non-alcoholic fatty liver disease-related proteins. Such comparative studies will help to clarify whether BPF has a unique or enhanced binding pattern compared to alternatives, thereby providing a structural and energetic basis for understanding its relative toxicological efficacy. This approach will directly contribute to establishing the “structure-activity relationship” of bisphenol compounds and support more accurate risk assessment of BPF as a widely used BPA substitute. Molecular dynamics simulations also confirmed the stability of the interaction of SHBG and CYP2C19 proteins with the compound BPF. The mechanism of action between BPF and SHBG and CYP2C19 has shown high research value. However, the specific mechanism of how BPF induces NAFLD by regulating SHBG and CYP2C19 protein expression needs to be further studied. The limitations of this study: The ADMET predictions and network toxicology methods used in this study are all based on theoretical predictions derived from known data and algorithms. These prediction results provide a strong direction for the formulation of hypotheses, but their biological authenticity must be verified through subsequent in vitro and in vivo experiments. The accuracy of this model is limited by the completeness and quality of the training data, and it cannot fully simulate the complex regulatory networks within the biological system.

## 5. Conclusion

In this study, network toxicology and molecular docking technology were used to explore the potential toxic targets and mechanisms of BPF in NAFLD, and SHBG and CYP2C19 genes that may play key roles were screened, which preliminarily revealed the causal relationship between BPF and NAFLD and its complex molecular mechanism. BPF is widely used in various places and objects in our daily life, and the harm it brings cannot be ignored. Therefore, it is essential to further explore the mechanism by which BPF affects the progression of NAFLD in subsequent studies.

## Supporting information

S1 Fig Analyze the BPF toxicity model using the ADMETlab 3.0 software. (PDF)

S2 Fig The specific grid center coordinates and box-shaped area dimensions of BDF, SHBG and CYP2C19. (PDF)

S3 Fig The binding mode and binding free energy of SHBG with the known ligand dihydrotestosterone are presented. (PDF)

S4 Fig The different docking conformations of SHBG and Bisphenol F and the corresponding scores are presented. (PDF)

S5 Fig The different docking conformations of CYP2C19 and Bisphenol F and the corresponding scores are presented. (PDF)

S1 Table Through the GEO2R analysis, differentially expressed genes (DEGs) were identified. (XLS)
