# Supplementary material for: Analysis of molecular targets and mechanisms of Bisphenol F (BPF)-induced non-alcoholic fatty liver disease (NAFLD) based on network toxicology and molecular dynamics
Source: PLoS One. 2026 Jun 18;21(6):e0351730. doi: 10.1371/journal.pone.0351730 (PMC13278446; doi:10.1371/journal.pone.0351730)
Supplement: S2 Fig — (PDF) [file pone.0351730.s002.pdf]

```
receptor = SHBG.pdbqt

ligand = BisphenolF.pdbqt

center_x = 27.252

center_y = 14.415

center_z = 37.573

size_x = 40.5

size_y = 47.25

size_z = 40.5

out = BisphenolF_out.pdbqt
```

```
receptor = CYP2C19.pdbqt

ligand = BisphenolF.pdbqt

center_x = -63.212

center_y = 10.833

center_z = -31.819

size_x = 47.25

size_y = 47.25

size_z = 47.25

out = BisphenolF_out.pdbqt
```
