# Supplementary figures and images for: Analysis of molecular targets and mechanisms of Bisphenol F (BPF)-induced non-alcoholic fatty liver disease (NAFLD) based on network toxicology and molecular dynamics
Source: PLoS One. 2026 Jun 18;21(6):e0351730. doi: 10.1371/journal.pone.0351730 (PMC13278446; doi:10.1371/journal.pone.0351730)

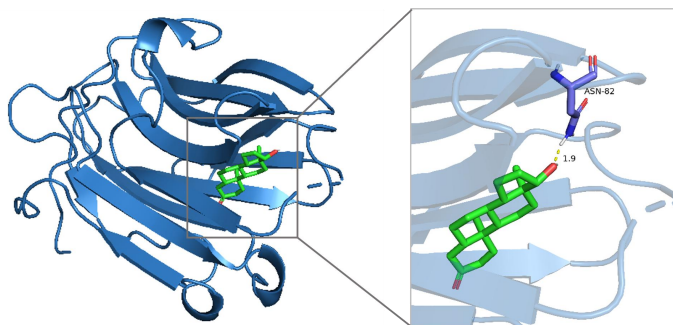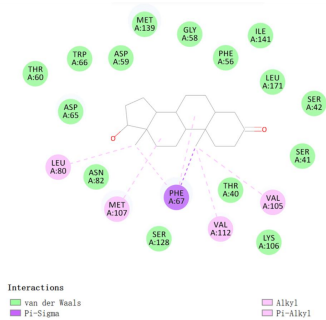

Supplement: S3 Fig — (PDF) [file pone.0351730.s003.pdf]

| mode                    | affinity   | dist from best mode |          |
|-------------------------|------------|---------------------|----------|
|                         | (kcal/mol) | RMSD l.b.           | rmsd u.b |
| -----+-----+-----+----- |            |                     |          |
| 1                       | -8.0       | 0.000               | 0.000    |
| 2                       | -8.0       | 0.775               | 1.768    |
| 3                       | -8.0       | 0.756               | 5.704    |
| 4                       | -8.0       | 0.305               | 5.751    |
| 5                       | -7.7       | 1.881               | 2.543    |
| 6                       | -7.7       | 1.770               | 5.993    |
| 7                       | -7.7       | 1.815               | 5.781    |
| 8                       | -7.5       | 1.866               | 2.401    |
| 9                       | -6.2       | 3.107               | 5.644    |

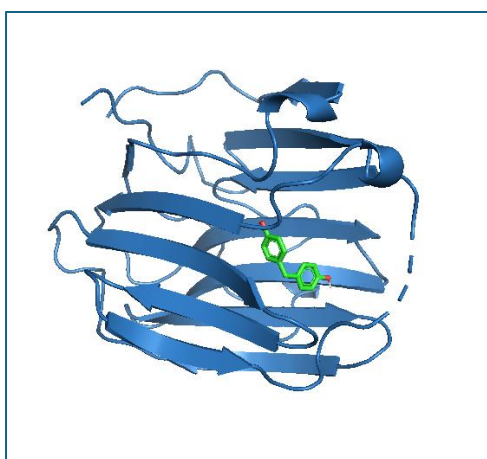

1

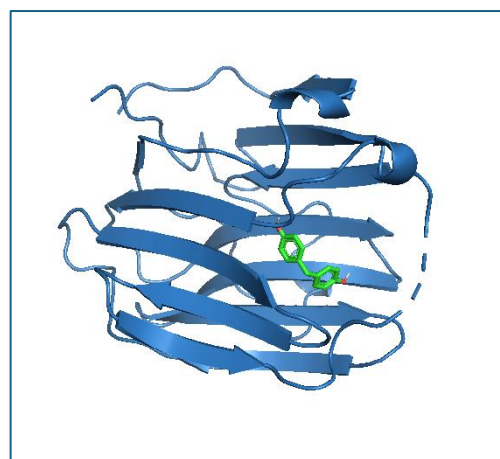

2

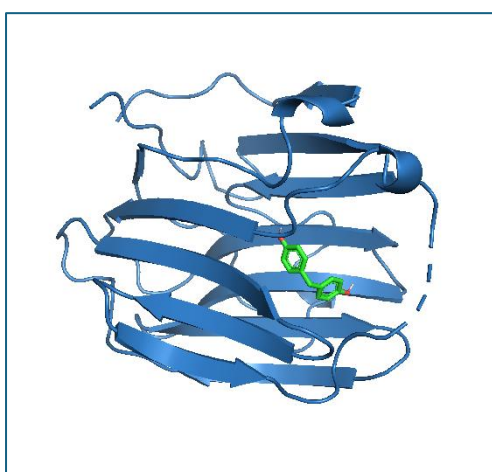

3

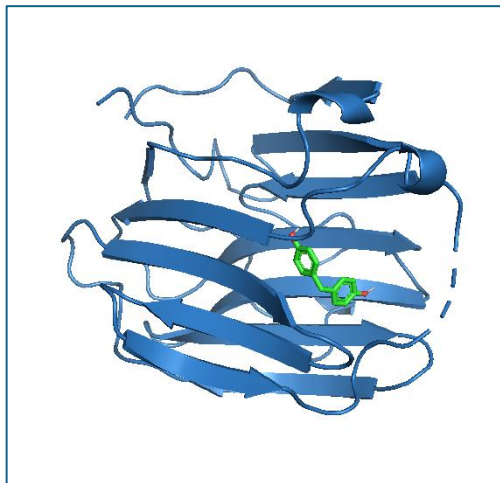

4

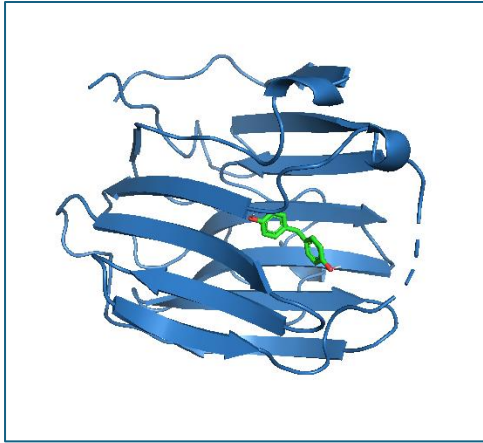

5

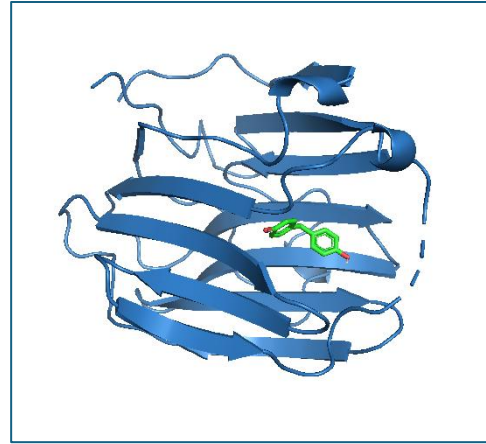

6

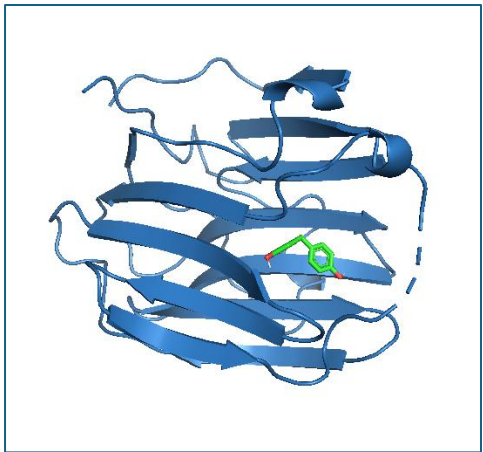

7

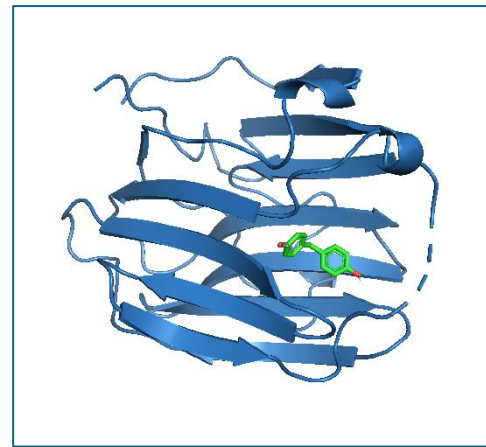

8

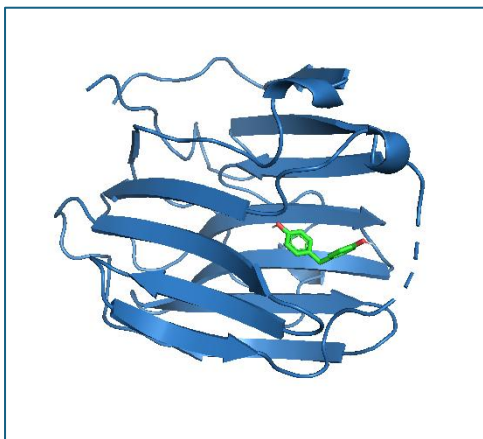

9

Supplement: S4 Fig — (PDF) [file pone.0351730.s004.pdf]

| mode              | affinity   | dist from best mode |          |
|-------------------|------------|---------------------|----------|
|                   | (kcal/mol) | RMSD l.b.           | rmsd u.b |
| -----+-----+----- |            |                     |          |
| 1                 | -7.8       | 0.000               | 0.000    |
| 2                 | -7.5       | 14.250              | 15.659   |
| 3                 | -7.5       | 14.244              | 15.758   |
| 4                 | -7.3       | 13.718              | 15.234   |
| 5                 | -7.3       | 13.213              | 14.822   |
| 6                 | -7.2       | 19.140              | 22.264   |
| 7                 | -7.2       | 19.045              | 21.709   |
| 8                 | -7.0       | 3.127               | 7.016    |
| 9                 | -6.9       | 17.460              | 18.978   |

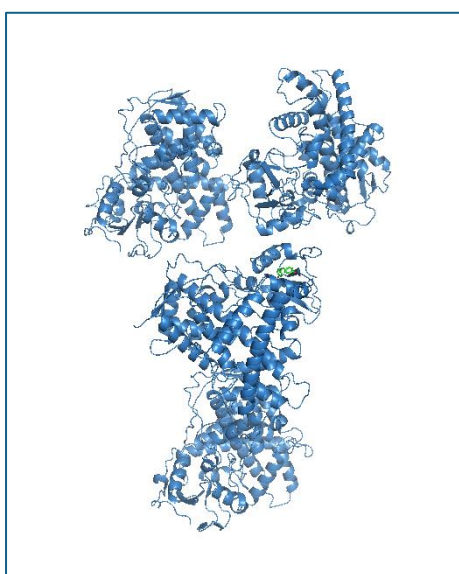

1

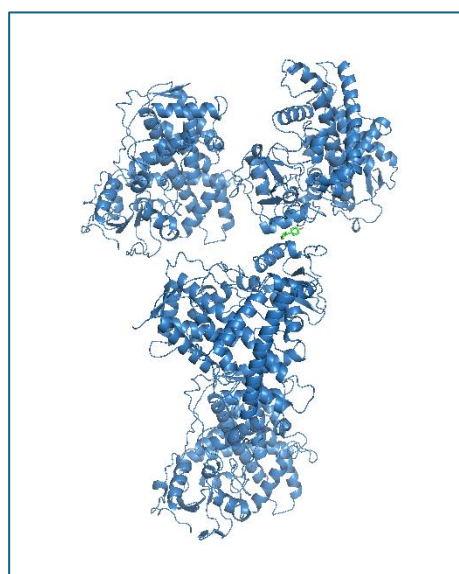

2

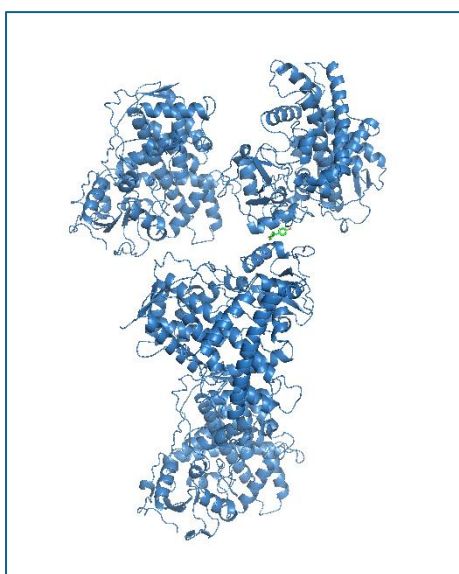

3

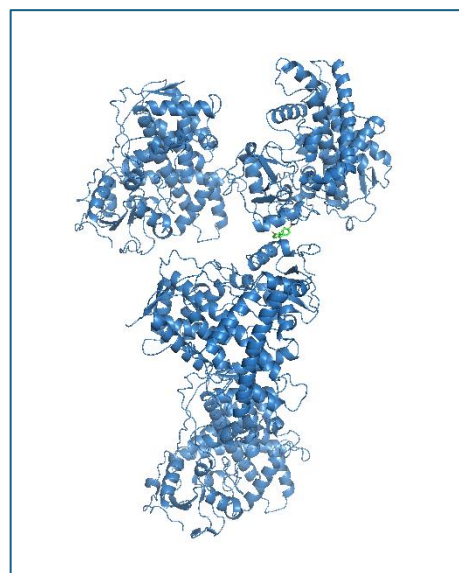

4

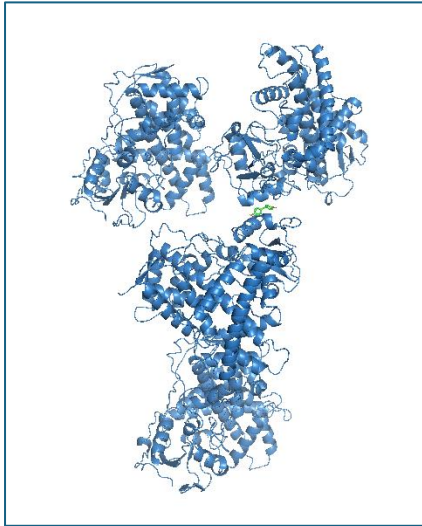

5

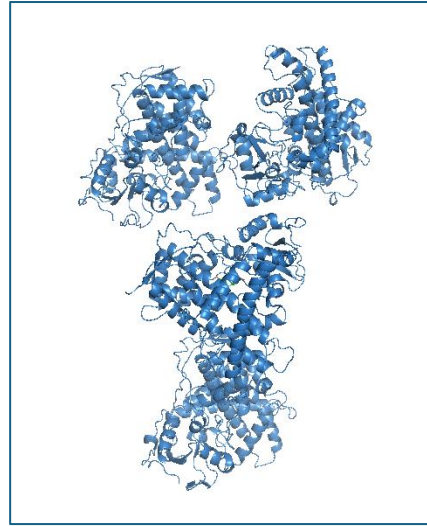

6

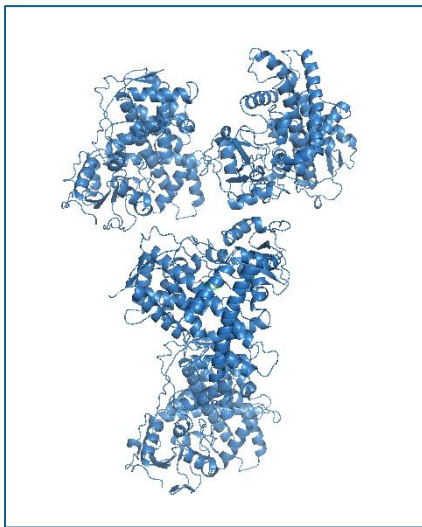

7

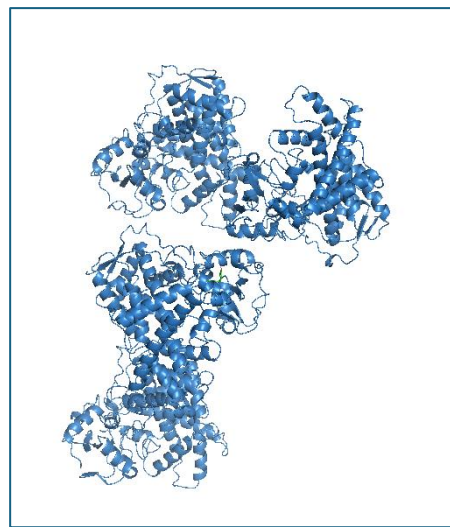

8

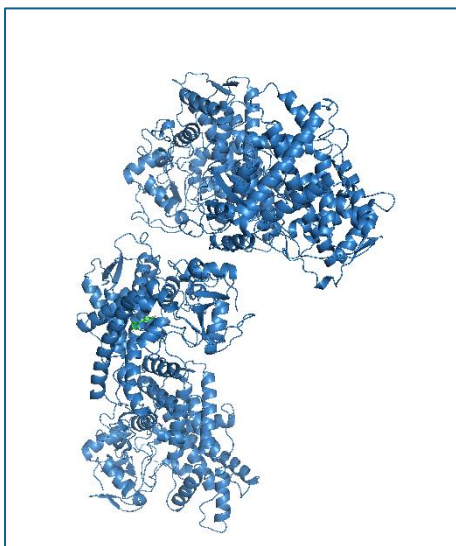

9

Supplement: S5 Fig — (PDF) [file pone.0351730.s005.pdf]
